# Developmental and familial predictors of adult cognitive traits in the European starling

**DOI:** 10.1016/j.anbehav.2015.07.002

**Published:** 2015-09

**Authors:** Daniel Nettle, Clare P. Andrews, Pat Monaghan, Ben O. Brilot, Thomas Bedford, Robert Gillespie, Melissa Bateson

**Affiliations:** a Centre for Behaviour and Evolution & Institute of Neuroscience, Newcastle University, Newcastle, U.K.; b Institute of Biodiversity, Animal Health & Comparative Medicine, University of Glasgow, Glasgow, U.K.; c School of Biological Sciences, Plymouth University, Plymouth, U.K.

**Keywords:** cognition, developmental stress, impulsivity, intelligence, learning, starlings, telomeres

## Abstract

In birds, there is evidence that adult cognitive traits can both run in families and be affected by early developmental influences. However, different studies use different cognitive tasks, which may not be measuring the same traits, and also focus on different developmental factors. We report results from a study in which we administered multiple cognitive tasks (autoshaping, discrimination learning, reversal learning, progressive ratio schedule, extinction learning and impulsivity) to a cohort of 34 European starlings, *Sturnus vulgaris*, for which several early developmental measures were available. The cohort consisted of siblings raised either apart or together, whose position in the size hierarchy of the rearing brood had been experimentally manipulated. We examined how the different cognitive measures covaried, the extent to which they ran in families, and which of the developmental factors predicted which of the cognitive outcomes. We found that discrimination and reversal learning speeds were positively correlated, as were breakpoint on the progressive ratio schedule and resistance to extinction. Otherwise, the cognitive measures were uncorrelated, suggesting that they reflected different underlying traits. All traits except discrimination and reversal learning speed ran in families to a substantial extent. Using a model selection approach, we found evidence that natal brood size and developmental telomere attrition (the extent to which the birds' erythrocyte telomeres shortened in early life, an integrative measure of developmental stress) were related to several adult cognitive measures. Results are discussed with respect to the best way of measuring avian cognitive abilities, and the utility of developmental telomere attrition as a predictor of adult outcomes.

## Highlights

• Cognition in birds may be influenced by familial and developmental factors.
• We administered cognitive tasks to 34 cross-fostered starlings from eight families.
• Tasks were uncorrelated, except for two measures of learning and two of persistence.
• Performance on most tasks ran in families.
• Natal brood size and early life telomere loss were associated with cognition.

Evidence from several different taxa, including humans, suggests that conditions experienced during early development can influence cognitive traits in adulthood (Avital, Ram, Maayan, Weizman, & Richter-Levin, 2006; Frankenhuis & de Weerth, 2013; Nowicki, Searcy, & Peters, 2002; Oomen et al., 2010; Sampson, Sharkey, & Raudenbush, 2008). In some cases, developmental adversity is associated with impaired adult cognitive abilities. For example, three studies have found that patterns of early growth are related to learning speed in zebra finches, *Taeniopygia guttata* (Bonaparte, Riffle-Yokoi, & Burley, 2011; Brust, Krüger, Naguib, & Krause, 2014; Fisher, Nager, & Monaghan, 2006).

In other cases, adverse developmental conditions may induce adaptive shifts, rather than impairments, in cognition (Frankenhuis & de Weerth, 2013; Oomen et al., 2010). In a recent study in European starlings, *Sturnus vulgaris* (Bateson, Brilot, Gillespie, Monaghan, & Nettle, 2015), we found that birds that had experienced more developmental telomere attrition (an integrative measure of developmental stress) had a stronger preference for immediate over delayed rewards as adults. We argued that the cognitive performance of the birds that had experienced poor early conditions was not worse, but simply reflected different priorities. This was borne out by the fact that in these studies there was no relationship between developmental telomere attrition and measures of associative learning speed that were incidentally generated during training. However, the possibility that the effects we observed indicated subtly reduced cognitive ability cannot be excluded.

While there has been a recent burgeoning of interest in measuring individual differences in cognition in birds (e.g. Boogert, Giraldeau, & Lefebvre, 2008; Cauchard, Boogert, Lefebvre, Dubois, & Doligez, 2013; Cole, Morand-Ferron, Hinks, & Quinn, 2012; Keagy, Savard, & Borgia, 2009), different studies use different cognitive tasks. Thus, it is hard to be sure exactly which aspect of cognition is being captured in each study, especially since some tasks may reflect boldness, neophobia or food motivation more than cognitive ability (Rowe & Healy, 2014; Templeton, Laland, & Boogert, 2014). Where studies have administered multiple tasks to the same individuals, they have mostly found intercorrelations between tasks to be weak or absent, suggesting that multiple independent capacities are being tested (Boogert, Anderson, Peters, Searcy, & Nowicki, 2011; Isden, Panayi, Dingle, & Madden, 2013; Keagy, Savard, & Borgia, 2011; Keagy et al., 2009). A notable exception is the positive correlation between trials to acquire a discrimination and trials to acquire its reversal observed in a recent study of song sparrows, *Melospiza melodia* (Boogert et al., 2011). The relationships of the many other commonly used cognitive tasks to learning speed as measured using discrimination and reversal learning are not well established.

We had access to a cohort of captive European starlings whose position in the within-brood size hierarchy had been experimentally manipulated, and whose developmental histories were very well characterized (Nettle et al., 2015). Quartets of chicks had been taken from their natal nests soon after hatching, and cross-fostered, two to a nest where they were slightly larger than the other chicks (advantaged treatment), and the other two to a nest where they were slightly smaller (disadvantaged treatment). The quartets were likely to have consisted of genetic siblings, since intraspecific brood parasitism affects only a minority of nests in the European starling (Pinxten, Eens, & Verheyen, 1991), and no nests were used where the clutch increased by more than one egg per day or the eggs obviously varied in colour. At posthatching day 12, the chicks were brought into captivity and kept in uniform conditions to adulthood. As we have reported previously, the developmental treatment had no effect on their growth curves, but the disadvantaged birds showed greater telomere attrition in early life than their advantaged siblings (Nettle et al., 2015).

The present paper reports the results of experiments conducted when the birds were between 5 and 13 months old, in which we measured, under standardized conditions, performance on a battery of cognitive tasks (see below for description). Our main aims in doing so were the following. First, we sought to investigate the extent to which performance on different tasks covaried; in particular, we were interested in whether discrimination and reversal learning speed were positively correlated, and which other tasks, if any, would also correlate with discrimination and reversal learning speed. Second, we sought to investigate which, if any, of the different measures of early developmental conditions was best at predicting adult cognitive performance. Finally, our design provided an opportunity to examine the extent of natal family influences on cognitive traits, as well as the impact of rearing nest. We found modest familial effects on impulsivity in our previous study (Bateson et al., 2015). By contrast, Bonaparte et al. (2011) found that familial effects on speed to acquire a conditioned discrimination in their zebra finches were trivial. This may suggest that different cognitive measures show different degrees of familial patterning.

Our battery of tasks included autoshaping, discrimination learning, reversal learning, progressive ratio schedule, extinction learning and an impulsivity measure. Autoshaping (Brown & Jenkins, 1968) exploits Pavlovian conditioning. A novel stimulus is repeatedly paired with the delivery of a food reward. The performance measure is the number of trials required before the subject begins to direct an appetitive response at the stimulus. Speed of autoshaping is conventionally interpreted as a measure of reinforcement learning (Markou et al., 2013). However, Feenders and Bateson (2013) concluded that individual differences in speed of autoshaping in starlings may primarily reflect individual differences in neophobia, rather than learning ability.

Discrimination learning involves pairing two arbitrary stimuli such as colours with different reward values (in some versions of the task, one stimulus is rewarded while the other is not; in ours, one was rewarded immediately and the other only after a delay, reducing its value to the starlings). The measure of learning speed is the number of trials required to acquire a preference. Discrimination learning is a relatively pure measure of associative learning ability, since neophobia or boldness can be eliminated as sources of variation. Reversal learning involves reversing the contingencies of the two stimuli once a discrimination has been acquired; the measure is the number of trials required until the subject reverses its previous preference.

In a progressive ratio schedule (Hodos, 1961), the number of instrumental responses required to release a reward is progressively increased. The measure is the breakpoint, the point at which the subject ceases responding. Progressive ratio schedule breakpoint is generally taken as a measure of incentive motivation rather than cognitive ability (Hodos, 1961; Kirkpatrick, Marshall, Smith, Koci, & Park, 2014; Markou et al., 2013); that is, the more motivated by the reward the subject is, the higher the breakpoint will be.

Extinction learning reflects how rapidly an individual ceases to respond to a stimulus that has previously been rewarded but no longer is. Delayed extinction in individuals that have experienced early life adversity has been observed in rodents and nonhuman primates, in which it has been interpreted as resulting from a maladaptive deficit in behavioural inhibition (Beauchamp & Gluck, 1988; Jones, Marsden, & Robbins, 1991).

Finally, we measured impulsivity using an adjusting-delay procedure (Mazur & Biondi, 2009), similar to our previous study (Bateson et al., 2015). Here, impulsivity is conceptualized as the extent to which a reward is devalued by having to wait additional time to receive it. Birds are trained that one stimulus produces a small reward after a short fixed delay, while another stimulus produces a large reward after a long adjustable delay. The length of the adjustable delay is titrated to estimate the point at which the individual is indifferent between the two options. An individual whose indifference point is at a relatively short adjustable delay discounts delay to reward more steeply, and hence is more impulsive, than an individual whose indifference point is at a longer adjustable delay.

Our measures of early life conditions included, in addition to developmental treatment, natal brood size, early growth rate and telomere length change from day 3 to day 12. Natal brood size is likely to reflect the quality or current condition of the genetic mother, with higher-quality or better-condition females laying larger clutches (Christians, Evanson, Aiken, & Aiken, 2001). Early growth rate was a key factor in previous studies of developmental effects on cognition in zebra finches (Bonaparte et al., 2011; Brust et al., 2014; Fisher et al., 2006). Telomere attrition during development is emerging as a useful integrative marker of developmental stress exposure in birds (Boonekamp, Mulder, Salomons, Dijkstra, & Verhulst, 2014; Herborn et al., 2014; Nettle, Monaghan, Boner, Gillespie, & Bateson, 2013), and it was a key predictor of adult cognition in a previous study (Bateson et al., 2015). We also considered adult body condition (defined as weight for skeletal size at time of completing the tasks) as a predictor of the cognitive variables. While not directly a developmental measure, adult body condition is a useful indicator of current state.

## Methods

### Subjects and Husbandry

Subjects were 37 European starlings (14 female, 23 male), hatched in the wild in May 2013, and taken into captivity before fledging. One bird had to be euthanized as a result of an accident in October 2013 (no measures completed) and one other died of unknown cause in June 2014 (all measures but impulsivity completed, and so sample size for analyses involving impulsivity is one bird smaller than other measures). When not in experimental procedures, birds were housed in groups of up to 20 in two indoor aviaries (215 × 340 cm and 220 cm high; ca. 18 °C; 40% humidity; 15:9 h light:dark), provided with environmental enrichment (rope perches, boxes for cover, wood shavings, water baths), clean drinking water and ad libitum food. Diet in aviaries and experimental cages consisted of chick crumbs (Special Diet Services Ltd, Witham, U.K., ‘HPS’) supplemented with cat biscuits (Royal Canin Ltd, royalcanin.co.uk, ‘Fit’), dried insect food (Orlux insect patée), live mealworms and fruit. The birds were maintained in nonbreeding condition at all times by the use of an unchanging cycle of 15 h days.

### Developmental Manipulation and Measures of Early Development

The birds were subjected to a developmental manipulation, which has been described in full elsewhere (Nettle et al., 2015). Briefly, on posthatching day 2, quartets of siblings matched for weight were cross-fostered, two to a nest where they were (mean + SD) 4.9 + 1.9 g larger than the average of the other nestlings (advantaged treatment) and the other two to a nest where they were 4.8 + 2.2 g smaller than the average of the other nestlings (disadvantaged treatment). Experimentally composed broods always consisted of five or six chicks in total, with brood size the same for all birds from the same natal nest. The birds were left in their host nests until day 12, whereupon they were brought into the laboratory, the two treatment groups mixed, and the birds hand-reared to independence. The groups did not differ in weight at any time during the developmental manipulation.

The birds were blood-sampled on day 3 and day 12. Erythrocyte telomeres were measured by qPCR, normalized against a known single-copy gene (full details in Nettle et al., 2015). This produces a measure called the T/S ratio, which represents the relative abundance in the bird's DNA of the telomeric sequence. Owing to some failed assays, telomere measures were available for only 34 birds (17 from each developmental treatment). For the rest of the paper, we consider these 34 birds only (33 birds for impulsivity). T/S ratios reduced significantly from day 3 to day 12; however, there was substantial variation in the extent of the reduction (Nettle et al., 2015). To represent developmental telomere length change with a single number, we used Verhulst et al.'s *D* (Verhulst, Aviv, Benetos, Berenson, & Kark, 2013), an index that corrects for regression to the mean. We calculated *D* so that a more negative number indicates greater telomere attrition. The *D* index was highly correlated with the simple difference between the day 3 and day 12 T/S measurements (*r*_32_ = 0.96, *P* < 0.01), and all results reported hereafter were extremely similar if the simple difference was used instead. Despite incorporating a correction for regression to the mean, *D* was significantly negatively correlated with day 3 T/S ratio (*r*_32_ = −0.42, *P* = 0.01). This is a commonly observed pattern (Verhulst et al., 2013). Further correcting *D* for day 3 T/S ratio produced very similar results to those reported below.

Our measure of variation in early growth rate was based on the residual from the regression equation predicting day 12 weight from day 7 weight for all 34 chicks. Day 12 and day 7 are both within the period of linear growth. Thus, a positive residual from this equation indicates that the bird grew relatively fast through this period, while a negative residual indicates that it grew relatively slowly. The growth rate measure was positively correlated with absolute weight at day 12 (*r*_32_ = 0.70, *P* < 0.01), and was also significantly related to adult skeletal size as indicated by tarsus length at day 24 (*r*_32_ = 0.37, *P* = 0.03). Adult body condition for the study was measured on each entry into the experimental cages, and calculated using the residual from the best-fitting equation relating day 24 weight to skeletal size (as estimated by day 24 tarsus length) for this cohort of birds.

### Experimental Cages

Birds completed the cognitive tasks in individual home cages (100 × 45 cm and 45 cm high) containing an operant panel of three illuminable pecking keys and a feeder trough connected to a pellet dispenser delivering 45 mg grain-based rodent pellets, as described in Feenders and Bateson (2013). The panels were controlled remotely using the Whisker Experimental Control system (Cardinal & Aitken, 2010), and cognitive tasks were programmed in Microsoft Visual Basic 5.0 (Microsoft Corporation, Redmond, WA, U.S.A). Temperature and lighting conditions were the same in the experimental room as in the aviary. Birds were run in replicates of eight consisting of two natal families.

### Experimental Procedures

#### General

**Figure 1:**
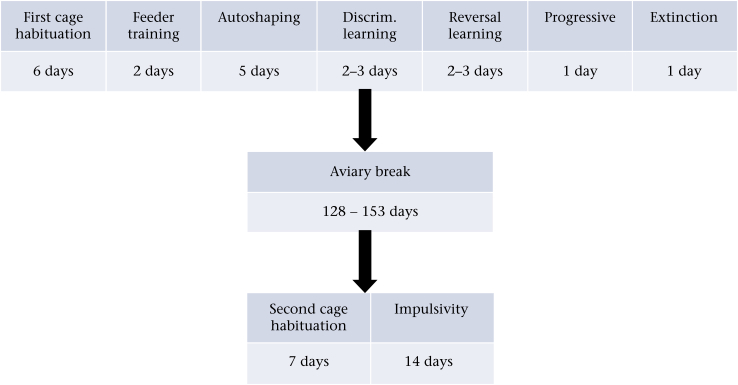
Timeline of the experiment, with typical durations of each phase.

Testing took place between September 2013 and June 2014. Each replicate of eight birds completed two periods in the experimental cages (see Fig. 1). In the first, they completed cage habituation, feeder training, autoshaping, discrimination and reversal learning, progressive ratio and extinction. They then had a break of 128–153 days during which they were mostly in the aviaries, although all birds did complete some unrelated short experiments during this time. In the second period, they completed a further cage habituation and the impulsivity task. The replicates were run in the same order for the two periods. Because replicates of birds were run sequentially, birds were different ages at time of testing. However, age at testing was unrelated to developmental treatment. Moreover, there were no significant differences in performance on any of the cognitive tasks with age (data not shown).

Experimenters were never present in the experimental room during testing. Birds were deprived of their food and baths at approximately 1700 hours each day, and cognitive tasks began automatically at 0730 hours. At 1230 hours, ad libitum food and baths were provided. The birds were then undisturbed until 1700 hours. Birds were weighed on entering the experimental cages and on release from them. They lost an average of 5.76 g (6.9% body weight) during the first period in experimental cages, and 3.64 g (4.6% body weight) during the second period. This weight loss was in line with previous studies (Feenders & Bateson, 2013).

#### First cage habituation

To habituate to being in cages and eating rodent pellets, the birds were given 1 week prehabituation in a separate room, initially caged in pairs, and subsequently on their own. By the end of the week, all birds were consuming pellets.

#### Feeder training

Birds were moved to the experimental cages at 1300 hours. At 1700 hours, ad libitum food was removed and the birds were left with 5 g of pellets in the feeder trough. At 0900 hours the next day, the trough was inspected. If the bird had foraged from the trough, a feeder training program was initiated; if not, initiation was delayed for 1 h. The program delivered two pellets repeatedly for 60 trials, with an average intertrial interval (ITI) of 200 s (uniform distribution from 150 s to 250 s). The feeder trough was illuminated for 3 s while the pellets were delivered. Feeder training was continued until all birds were consuming pellets as soon as they were delivered.

#### Autoshaping/shaping

In each trial, the centre key was illuminated amber for 15 s, ending with the delivery of two pellets. The autoshaping speed measure was the number of the trial on which the bird first pecked the lit key. Pecking the key truncated the illumination interval and immediately released the reward. There were 80 trials per day with an ITI of 200 s. If the bird had pecked the key within the interval on at least half the trials on the second day, the reward delivery was made conditional on the bird pecking within the interval; if not, the reward delivery remained unconditional until this criterion had been met. Once the bird had moved to conditional delivery, it required 3 successive days on which it pecked in >80% of trials. Extra days were added where necessary until this criterion was met.

#### Discrimination learning

To ensure that the subject was attending, discrimination learning trials commenced with the centre amber key being illuminated. Once this initiation key was pecked, the trial proper began. In forced trials, one of the side keys (left or right, randomized) was illuminated either green or red. Pecking the key if green began a fixed interval of 2 s until one pellet was released, whereas pecking the key if red began a fixed interval of 10 s until one pellet was released. In choice trials, both of the side keys were illuminated, one red and one green (sides randomized). Pecking either one caused the other side key to de-illuminate, and began the fixed interval for one pellet associated with the chosen colour. Discrimination trials were presented in blocks of 10 (16 blocks per day), with six choices and four forced trials in each block, in pseudorandom order, and an ITI of 90 s. The purpose of the forced trials was to ensure that all subjects gained information about the value of both options. The criterion for progression to the next task was more than 80% choices for the short delay on a day on which more than 60 trials had been completed. The measure of learning speed was the number of blocks the subject required until it chose the colour associated with the short interval at least five out of six times for two successive blocks.

#### Reversal learning

The procedure for reversal learning was exactly as for discrimination learning, but the assignment of intervals to colours was reversed. The criterion for successful acquisition and the measure of learning speed were the same as for discrimination learning.

#### Progressive ratio schedule

The progressive ratio schedule task used the centre key illuminated amber. The number of pecks required to release one pellet was initially set at one, then increased by five with every trial successfully completed (ITI 100 s). Subjects had to complete the required number of pecks within 20 min. If unsuccessful, the same ratio was repeated. The performance measure was the highest ratio successfully completed. This task was run for a single 5 h session.

#### Extinction learning

The extinction learning task used the centre key illuminated amber, which the subject had been reinforced for pecking through all previous tasks. It consisted of a single session of 160 trials (ITI 100 s). The key was illuminated for up to 15 s. For the first 10 trials, pecking the lit key was reinforced with one pellet. For the remaining trials, there was no reinforcement. The measure was the number of trials (out of 160) on which the subject pecked the key, and hence a higher number represents slower extinction.

#### Second cage habituation period

To rehabituate the birds to cages and to eating pellets, the birds were initially pair-housed in the experimental cages for 3 days while gradually shifting to a diet of pellets. Once the pellets were reliably being eaten (minimum 3 days), the birds were separated to their individual cages for 2 more days. They were then given 1 day of autoshaping to reinstate key pecking. Birds progressed to the impulsivity task if they succeeded in >80% of trials; where this was not met, a second day was added.

#### Impulsivity

In impulsivity forced trials, pecking a lit centre amber initiation key began a side key (side randomized) flashing (0.7 s on, 0.3 s off) either red or green. Once the flashing key was pecked, it became constantly illuminated for a preprogrammed delay. The first peck after the end of the delay produced a reward. One colour (counterbalanced across families) produced one pellet after a delay of 3 s, whereas the other produced two pellets after an adjustable delay (see below). In impulsivity choice trials, following the pecking of the initiation key, a red and green key (sides randomized) were presented simultaneously. Pecking either one caused the other to be de-illuminated and began the corresponding delay. Impulsivity trials (up to 120 per day) were presented in blocks of four (one forced fixed delay, one forced adjustable delay, two choices) in pseudorandom order. If the bird chose the adjustable delay option in both choice trials, the adjustable delay was increased by 1 s for the next block, whereas if the bird chose the fixed delay option in both trials, the adjustable delay was decreased by 1 s. The ITI was adjusted every trial so that ITI plus the programmed delay equalled 150 s. The adjusting delay was initialized at 3 s on the first day, and its duration at the end of one day was used to start the next. All birds completed 1400 trials. The measure of impulsivity was the mean value of the adjusting delay once the first 200 trials had been excluded. Hence, a higher value indicates a less impulsive individual.

### Statistical Analysis

Data were analysed using R (R Core Development Team, 2013). Autoshaping speed showed a highly right-skewed distribution and was logged for analysis. To address the aim of establishing the covariation between the different cognitive measures, we calculated and report their correlation matrix. (There was insufficient shared variance in this matrix to reduce the data using principal components analysis.) To examine familial and shared nest influences on the cognitive measures, we performed a variance partition analysis. This involved fitting a model with nested random effects of host nest within natal nest, and no fixed effects other than the intercept (package lme4, restricted maximum likelihood estimation). This allowed us to estimate the proportion of variation that is attributable to natal nest, to host nest within a natal nest, and to between-individual variation and measurement error. The importance of natal nest and host nest components was assessed by examining the change in the corrected Akaike information criterion (AICc) when the random effect under consideration was included in the model.

For the analysis of which developmental factors predict adult cognitive performance, we had several possible fixed predictors (natal brood size, developmental treatment, growth, developmental telomere length change, adult body condition). We used a model selection/model averaging approach (Symonds & Moussalli, 2010). We investigated all 32 possible models (the intercept-only model plus the 31 possible additive models containing some or all of the five fixed predictors), and retained as our candidate set of models all those within 2 AICc units of the best-fitting model (see Appendix for tables of models). The random effects structure for each of these models was guided by the results of the variance partition analysis. The package also provides an AICc weight for each model in the set. Using these weights, we produced a combined AICc weight for each fixed predictor under consideration, which can be considered as a measure of the strength of support for that particular predictor being included in the model. We also report parameter estimates and 95% confidence intervals for each predictor included in any of the candidate models. These are based on averaging the models in the candidate set (using package aicmodavg and maximum likelihood estimation).

### Ethical Note

The birds were taken from the wild under Natural England licence 20121066 and the research completed under Home Office licence PPL60/4073, with approval of the local ethical review committee at Newcastle University. All experimental procedures adhered to ASAB/ABS Guidelines for the Use of Animals in Research. One chick of 48 that we cross-fostered in 2013 died between cross-fostering and the next morning; this is no greater than the expected rate of mortality this early in life. All other cross-fostered chicks gained weight between before transplantation and the next morning, suggesting rapid recovery from transport and acceptance in host nests. The developmental stress created by the experimental manipulation is likely to have been within the natural range experienced by wild starlings. Two disadvantaged and three advantaged birds died before day 12; this is in line with rates of mortality in undisturbed nests in our starling colony.

In captivity, all birds spent most of their time in groups in large, enriched aviaries. For the periods when individual caging was necessary, all birds had a period of ad libitum food and access to baths each day, and had visual and auditory contact with conspecifics. The maximum period of complete food deprivation during the experiments was 14 h and 30 min (1700–0730 hours). Starlings do not feed during the hours of darkness, and winter darkness in the natal area in Northumberland exceeds 15 h per day, coupled with lower temperatures than our laboratory. Thus, the regime of food deprivation was within a range that starlings would cope with routinely in the wild.

## Results

The raw data are available as Supplementary Material.

### Covariation of Cognitive Measures

**Figure 2:**
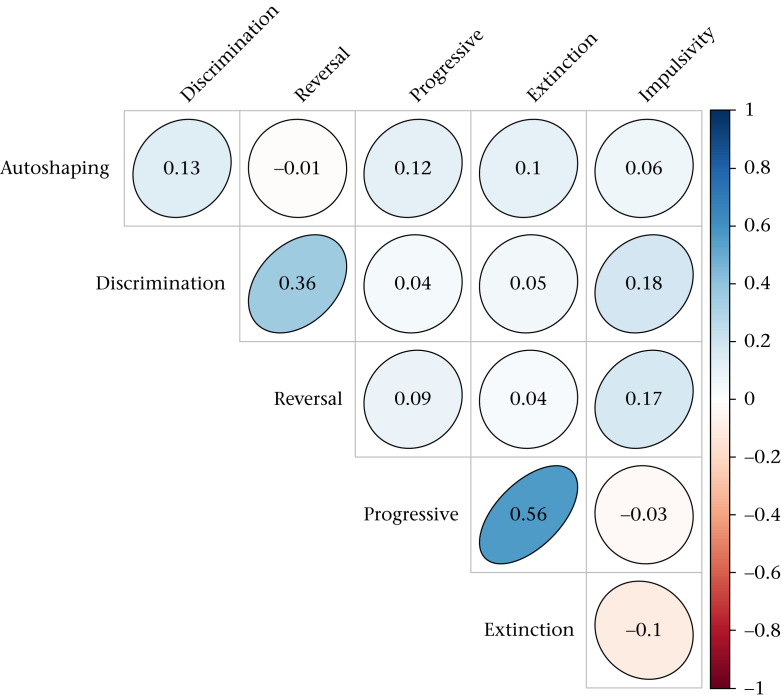
Correlation coefficients between the six cognitive measures. Depth of shading and shape of the ellipse represent the magnitude of the correlation. The only correlations significant at *P* < 0.05 against the null hypothesis that the true correlation is zero are Discrimination/Reversal and Progressive/Extinction.

The correlations between the six cognitive measures are shown in Fig. 2. The only substantive correlations were between discrimination and reversal learning speed, and between progressive ratio breakpoint and extinction. The relationship between discrimination and reversal learning speeds was positive, meaning that birds that took more blocks to acquire a discrimination also took more blocks to acquire the reversal. The relationship between progressive ratio breakpoint and extinction was also positive; birds that completed a higher ratio also continued responding for more trials once pecking was no longer reinforced.

### Variance Partition Analysis

**Figure 3:**
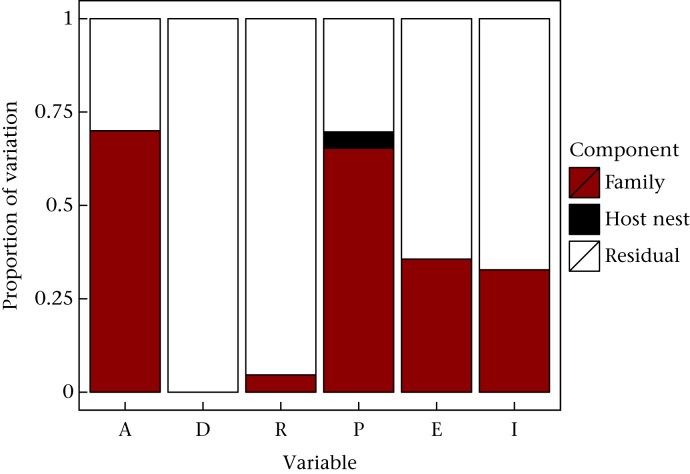
Components of variation (natal family, host nest, residual) for each of the six cognitive measures. A: logged autoshaping speed; D: discrimination speed; R: reversal; P: progressive ratio breakpoint; E: extinction; I: mean adjusting delay in impulsivity task.

For each of the six cognitive measures, we performed a variance partition analysis to decompose the variation accounted for by natal nest, shared host nest and the residual, which reflects individual differences within a sibling pair from the same treatment as well as measurement error. Figure 3 summarizes the variance components for each measure. Logged autoshaping speed and progressive ratio breakpoint showed large natal family components (ΔAICc = −12.16 and ΔAICc = −6.69), while extinction learning speed and impulsivity showed modest natal family components (ΔAICc = 0.04 and −1.01). Discrimination and reversal learning speed showed trivial or zero natal family components (ΔAICc = 2.41 and 2.32). The component due to shared host nest was in all cases zero or very close to zero (all ΔAICc > 2.39). In view of these results, we retained random effects of natal family for subsequent analyses for autoshaping, progressive ratio schedule, extinction and impulsivity, but not for discrimination or reversal learning.

### Developmental Predictors of Cognitive Measures

#### Autoshaping

For autoshaping, four models were retained in the candidate set (Appendix Table A1). All four contained developmental telomere length change. The effect of developmental telomere length change was negative (*B* = −2.20, 95% confidence interval, CI −3.25 to −1.15); that is, greater telomere attrition was associated with more trials required to autoshape. Three of the four candidate models also included natal brood size. The effect of natal brood size was also negative, with birds from smaller natal broods requiring more trials (*B* = −0.79, 95% CI −1.47 to −0.10). This effect appeared to be due to the birds from the smallest natal broods (four chicks) being slower to autoshape than all other birds. One of the four candidate models also included growth rate. The effect of growth rate tended to be negative, with birds that grew more slowly tending to require more trials (*B* = −0.16, 95% CI −0.16 to 0.03). Finally, one of the four candidate models included developmental treatment (*B* = −0.26, 95% CI −0.74 to 0.22).

#### Discrimination learning

Two candidate models were identified for discrimination learning speed, the intercept-only model and a model with body condition (Appendix Table A2). The association between body condition and discrimination speed tended to be positive, with relatively heavier birds taking more blocks (*B* = 0.15, 95% CI −0.12 to 0.43).

#### Reversal learning

*F*or reversal learning speed, two candidate models were retained (Appendix Table A3). Both of these contained natal brood size. The effect of natal brood size was negative, with birds from smaller natal broods requiring more blocks to learn the reversal (*B* = −1.32, 95% CI −2.44 to −0.22). One of the two candidate models contained early growth rate. The effect of early growth rate tended to be positive; that is, birds with relatively fast early growth tended to require more blocks to learn the reversal (*B* = 0.24, 95% CI −0.07 to 0.54).

#### Progressive ratio schedule

Four candidate models were identified for progressive ratio schedule breakpoint (Appendix Table A4). All four contained developmental telomere length change. The effect of developmental telomere length change was positive; birds that had experienced greater telomere attrition during development had lower breakpoints (*B* = 71.41, 95% CI 9.66 to 133.16). Two of the four candidate models contained natal brood size. The effect of natal brood size tended to be negative, with birds from larger natal broods having lower breakpoints (*B* = −24.86, 95% CI −52.59 to 2.86). Two models also contained developmental treatment. The effect of being from the disadvantaged treatment tended to be negative (*B* = −18.36, 95% CI −46.18 to 9.47).

#### Extinction learning

Four candidate models were identified for extinction learning speed, one of which was the intercept-only model (Appendix Table A5). Two of the models contained developmental telomere length change. The effect tended to be positive (birds that had experienced greater telomere attrition during development extinguishing in fewer trials; *B* = 37.26, 95% CI −3.44 to 77.97). Two models contained natal brood size. The effect tended to be negative (birds from larger natal broods extinguishing in fewer trials; *B* = −8.81, 95% CI −21.27 to 3.65).

#### Impulsivity

Five candidate models were identified, one of which was the intercept-only model (Appendix Table A6). Two candidate models contained early growth rate. The effect of early growth rate tended to be positive, with birds that grew faster having longer mean adjusting delays, and hence being less impulsive (*B* = 0.15, 95% CI −0.03 to 0.33). Two candidate models included adult body condition. The effect of adult body condition tended to be positive, with relatively heavier adults having longer mean adjusting delays (*B* = 0.05, 95% CI −0.04 to 0.13). One candidate model included developmental telomere length change. The effect tended to be positive, with birds that experienced less attrition having higher mean adjusting delays (*B* = 1.04, 95% CI −1.29 to 3.37).

#### Summary of results

**Figure 4:**
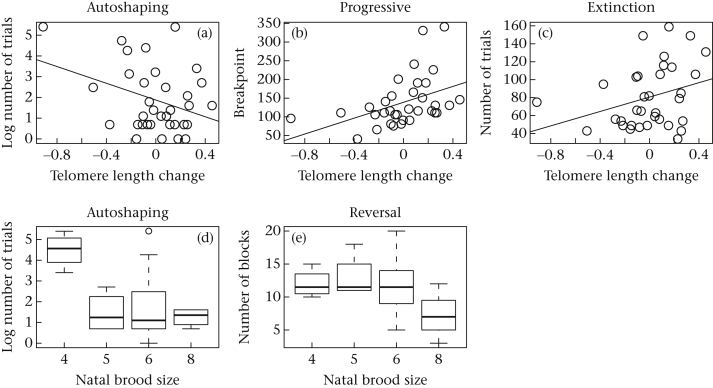
Illustrative plots for the most strongly-supported relationships between developmental predictors and adult cognitive measures. (a–c) The predictor is developmental telomere length change (Verhulst et al.'s *D*; more negative value equals more attrition); (d, e) the predictor is natal brood size.

Table 1 summarizes the findings with regard to predictor variables for each of the cognitive traits, and Fig. 4 displays plots illustrating each of the relationships that obtained either strong or moderate support.

## Discussion

To investigate the relationships among common measures of cognitive performance, and to examine their familial and developmental predictors, we administered a battery of cognitive tasks to a cohort of starlings of known developmental history, containing siblings raised both together and apart. The pattern of covariation of our measures was consistent with previous findings that, although different cognitive measures in birds are largely independent of one another (Boogert et al., 2011; Isden et al., 2013; Keagy et al., 2011, 2009), discrimination learning speed and reversal learning speed tend to be positively correlated (Boogert et al., 2011). It is possible that this correlation arises because an individual that takes more training to learn an association simply has more exposure to that association overall, and hence requires more exposure to its reversal to overcome it. None the less, discrimination and reversal learning are among the cleanest measures of learning speed, and hence of cognitive ability, since they are relatively independent of such factors as neophobia and boldness. This, and the fact that they are positively correlated, should make them a first choice in any attempt to measure avian cognitive abilities. Although both extinction and autoshaping might also be considered measures of speed of learning, neither of them correlated to any significant degree with discrimination or reversal learning, and neither did impulsivity or progressive ratio breakpoint. This suggests that inferences concerning birds' cognitive abilities from any single task, especially a task that does not involve learning a discrimination or reversal, may not be warranted.

We also found that breakpoint on a progressive ratio schedule and extinction learning were strongly related, with birds with a higher breakpoint also slower to extinguish. Progressive ratio schedule performance is generally taken as a measure of food motivation (Kirkpatrick et al., 2014; Markou et al., 2013), whereas slow extinction is generally taken as a measure of impairment in behavioural inhibition (Beauchamp & Gluck, 1988; Jones et al., 1991). Since these tasks were run on successive days, the null expectation would be that they would be negatively correlated, since a bird that continues responding for longer on a progressive ratio would accumulate more unreinforced responses over the course of the session, and hence be closer to extinction by the next day. However, all birds reinstated pecking in the 10 reinforced trials at the beginning of the extinction session. The positive correlation suggests instead the two tasks tap into a common trait, persistence in the pursuit of food. This trait appears unrelated to learning speed as measured by discrimination and reversal.

The variance partition analysis, a novel aspect of our study, showed that the cognitive traits differed in the extent to which they ran in natal families. Autoshaping speed and progressive ratio schedule breakpoint had large natal familial components. Extinction learning performance and impulsivity showed more modest natal family variation. Discrimination and reversal learning performance did not run in families to any detectable degree. This is consistent with the results of the only other study to directly test for family influence on discrimination learning in birds (Bonaparte et al., 2011). Why the different traits should differ so starkly in their familiality is not yet clear. We also cannot demonstrate from the current data whether the familial variance, where observed, is due to genetic influences, nongenetic parental effects or environmental influences that act before or around hatching. It is also possible that we somewhat underestimated the strength of genetic familial influences, if there was any undetected intraspecific brood parasitism in our sample. For none of the traits we studied did sharing a nest between day 2 and day 12 of life lead to increased cognitive resemblance in adulthood. This suggests that differences between parents in terms of state or experience either have little effect or, perhaps more plausibly, have different effects on different members of the brood.

We found evidence for developmental factors being related to adult cognitive performance, but there was a complex pattern with different developmental predictors relevant to different cognitive measures, as summarized in Table 1. The developmental factors with the strongest associations were developmental telomere attrition, which showed at least some evidence of association with four of the six cognitive traits, and natal brood size, again with at least some evidence for four of the six cognitive traits. Surprisingly, the developmental factor that we directly experimentally manipulated, position in the size hierarchy, showed no association with any cognitive measure. Overall, our results can be considered broadly consistent with previous research in that they suggest that early developmental influences have a measurable impact on aspects of adult cognition. However, just as our results differ from trait to trait, in many cases they differ in detail from the results of previous studies. Fisher et al. (2006) and Brust et al. (2014) found evidence for the importance of early growth or catch-up growth in predicting learning speed, whereas early growth was one of the less important predictors in our study. Relatively fast early growth showed weak evidence of association with slower autoshaping speed and greater impulsivity. For reversal learning, we found weak evidence that faster early growth was actually associated with slower speed to acquire the reversal. However, Fisher et al. and Brust et al. experimentally altered early growth through a dietary manipulation. By contrast, we only measured naturally occurring variation in growth. The year 2013 was an extremely favourable one in our starling colony and all observed growth rates were high. Thus, our sample includes only limited variation in growth, and no experimentally induced catch-up, which may account for the differential importance of early growth in our study compared to the previous ones.

Bateson et al. (2015) argued that developmental telomere attrition is a promising marker of the legacy of an individual's developmental history, because it integrates the consequences of multiple sources of developmental stress into a single somatic marker. Our study confirmed the view that adult cognitive traits are related to developmental telomere attrition, since this was the most strongly supported developmental influence across several measures. However, the specific associations described by Bateson et al. (2015), greater impulsivity being associated with greater developmental telomere loss and, independently, with lighter current body weight, were not clearly replicated. Although there was some evidence for both effects, in the same directions as in the previous study, the evidence was weak, and the confidence intervals for the parameters included zero in both cases. Why the impulsivity results should differ, given that the testing methods were very similar, is not clear. The birds were from different cohorts in different years, and there were some differences in the two experimental protocols. The developmental variable manipulated was different in the two studies, brood size in one case and position within the brood in the other. The time intervals of the telomere change measures were different in the two studies: Bateson et al. (2015) used a change measure over a longer window of early life (days 4–55), while in the current study, we measured change over just the period of the experimental manipulation (i.e. days 3–12). In this study, we found some evidence for the importance of early growth for impulsivity, with birds that grew relatively poorly tending to be more impulsive as adults. This is consistent with the general interpretation of impulsivity as a response to poor somatic state advanced by Bateson et al. (2015).

There are two possible interpretations of impacts of developmental factors on adult cognition (Frankenhuis & de Weerth, 2013). They may simply reflect pathology (poorer developmental conditions lead to a brain that functions less well), or they may represent some kind of adaptive plasticity, with individuals adopting the psychological priorities that are best suited to the phenotypic capacities with which development has left them. It is hard to adjudicate between these different interpretations for the effects we found, and the best interpretation may be different for different traits. A pathology interpretation would most obviously be supported had we found that discrimination and reversal learning were slower in individuals with less favourable developmental indicators. There were only fragmentary indications that this might have been so: birds from small natal broods (which may reflect low parental quality or poor parental state) acquired a reversal more slowly, were relatively slow to autoshape and to extinguish responding. Many of the other findings could equally well support a plasticity interpretation, for example that birds that experienced greater developmental telomere attrition were quick to give up in extinction or when facing increasing costs to obtain reward. Establishing whether these patterns are robust and replicable will require many more studies. Establishing whether they are adaptive will require different kinds of studies, where the individual differences in cognition are not just traced back to early development, but also followed forwards into naturalistic adult contexts, to relate them to fitness or fitness-relevant proxy outcomes.

## Figures and Tables

**Table A1:** Selected candidate models for (log) autoshaping speed

Model	*k*	AICc	ΔAICc	AICc weight	Loglikelihood
ΔTL+NBS	5	108.25	0.00	0.42	−48.05
ΔTL+Growth+NBS	6	109.56	1.31	0.22	−47.22
ΔTL	4	109.66	1.42	0.20	−50.14
Trt+ΔTL+NBS	6	110.11	1.86	0.16	−47.50

*k*: number of parameters in model; ΔAICc: change in AICc compared to best-fitting model (zero for best-fitting model itself); ΔTL: developmental telomere length change; NBS: natal brood size; growth: early growth rate; Trt: developmental treatment.

**Table A2:** Selected candidate models for discrimination learning speed

Model	*k*	AICc	ΔAICc	AICc weight	Loglikelihood
Intercept only	2	207.06	0.00	0.65	−101.34
Body condition	3	208.26	1.20	0.35	−100.73

*k*: number of parameters in model; ΔAICc: change in AICc compared to best-fitting model (zero for best-fitting model itself).

**Table A3:** Selected candidate models for reversal learning speed

Model	*k*	AICc	ΔAICc	AICc weight	Loglikelihood
NBS	3	184.17	0.00	0.51	−88.68
Growth+NBS	4	184.25	0.08	0.49	−87.44

*k*: number of parameters in model; ΔAICc: change in AICc compared to best-fitting model (zero for best-fitting model itself); NBS: natal brood size; growth: early growth rate.

**Table A4:** Selected candidate models for progressive ratio schedule breakpoint

Model	*k*	AICc	ΔAICc	AICc weight	Loglikelihood
ΔTL	4	377.83	0.00	0.33	−184.23
ΔTL+NBS	5	377.88	0.05	0.32	−182.87
ΔTL+Trt	5	378.95	1.11	0.19	−183.40
ΔTL+Trt+NBS	6	379.35	1.52	0.16	−182.12

*k*: number of parameters in model; ΔAICc: change in AICc compared to best-fitting model (zero for best-fitting model itself); ΔTL: developmental telomere length change; NBS: natal brood size; Trt: developmental treatment.

**Table A5:** Selected candidate models for extinction learning speed

Model	*k*	AICc	ΔAICc	AICc weight	Loglikelihood
Intercept only	3	342.24	0.00	0.31	−167.72
ΔTL	4	342.30	0.07	0.30	−166.46
ΔTL+NBS	5	342.87	0.64	0.23	−165.37
NBS	4	343.55	1.31	0.16	−167.08

*k*: number of parameters in model; ΔAICc: change in AICc compared to best-fitting model (zero for best-fitting model itself); ΔTL: developmental telomere length change; NBS: natal brood size.

**Table A6:** Selected candidate models for impulsivity

Model	*k*	AICc	ΔAICc	AICc weight	Loglikelihood
Intercept only	3	142.44	0.00	0.32	−67.81
Growth	4	142.65	0.21	0.21	−66.61
BC	4	144.04	1.60	0.14	−67.31
Growth+BC	5	144.37	1.93	0.12	−66.08
ΔTL	4	144.39	1.94	0.12	−67.48

*k*: number of parameters in model; ΔAICc: change in AICc compared to best-fitting model (zero for best-fitting model itself); growth: early growth rate; BC: body condition; ΔTL: developmental telomere length change.

**Table 1:** Summary of findings for predictors of adult cognitive variables

	Autoshaping speed	Discrimination learning speed	Reversal learning speed	Progressive ratio schedule	Extinction	Impulsivity
Strong support (AICc weight=1)	Developmental telomere length change^a^ (more attrition, slower autoshaping)		Natal brood size^a^ (smaller broods, slower to acquire)	Developmental telomere length change^a^ (more attrition, lower breakpoint)		
Moderate support (0.5< AICc weight <1)	Natal brood size^a^ (small natal brood, slower autoshaping)				Developmental telomere length change (more attrition, faster extinction)	
Weak support (AICc weight <0.5)	Early growth rate (slower early growth, slower autoshaping) Developmental treatment (advantaged birds, slower autoshaping)	Adult body condition (heavier birds slower to learn)	Early growth rate (faster early growth, slower to acquire)	Natal brood size (larger natal brood, lower breakpoint) Developmental treatment (disadvantaged birds lower breakpoint)	Natal brood size (larger natal brood, faster extinction)	Early growth rate (poorer early growth, more impulsive) Body condition (lighter birds more impulsive) Developmental telomere length change (more attrition, more impulsive)
No support	Adult body condition	Developmental treatment Developmental telomere length change Natal brood size Early growth rate	Developmental treatment Developmental telomere length change Adult body condition	Early growth rate Body condition	Developmental treatment Early growth rate Body condition	Developmental treatment Natal brood size

a 95% confidence interval for the parameter estimate does not include zero.
